# A Muscle Synergy-Inspired Adaptive Control Scheme for a Hybrid Walking Neuroprosthesis

**DOI:** 10.3389/fbioe.2015.00203

**Published:** 2015-12-21

**Authors:** Naji A. Alibeji, Nicholas Andrew Kirsch, Nitin Sharma

**Affiliations:** ^1^Department of Mechanical Engineering and Materials Science, University of Pittsburgh, Pittsburgh, PA, USA

**Keywords:** non-linear control, adaptive control, time-invariant synergies, functional electrical stimulation, hybrid neuroprosthesis

## Abstract

A hybrid neuroprosthesis that uses an electric motor-based wearable exoskeleton and functional electrical stimulation (FES) has a promising potential to restore walking in persons with paraplegia. A hybrid actuation structure introduces effector redundancy, making its automatic control a challenging task because multiple muscles and additional electric motor need to be coordinated. Inspired by the muscle synergy principle, we designed a low dimensional controller to control multiple effectors: FES of multiple muscles and electric motors. The resulting control system may be less complex and easier to control. To obtain the muscle synergy-inspired low dimensional control, a subject-specific gait model was optimized to compute optimal control signals for the multiple effectors. The optimal control signals were then dimensionally reduced by using principal component analysis to extract synergies. Then, an adaptive feedforward controller with an update law for the synergy activation was designed. In addition, feedback control was used to provide stability and robustness to the control design. The adaptive-feedforward and feedback control structure makes the low dimensional controller more robust to disturbances and variations in the model parameters and may help to compensate for other time-varying phenomena (e.g., muscle fatigue). This is proven by using a Lyapunov stability analysis, which yielded semi-global uniformly ultimately bounded tracking. Computer simulations were performed to test the new controller on a 4-degree of freedom gait model.

## 1 Introduction

Each year, approximately 5100 people in the USA alone are diagnosed with paraplegia due to a spinal cord injury (The National SCI Statistical Center, 2014), impairing their ability to walk again. Functional electrical stimulation (FES) and powered orthoses are two viable technologies that have the potential to restore the walking function in persons with SCI (Kralj and Bajd, 1989; Kobetic et al., 1997; Farris et al., 2011; Neuhaus et al., 2011; Esquenazi et al., 2012; del-Ama et al., 2014a; Ha et al., 2015). FES is a clinical technique in which the muscle is artificially stimulated with low level electrical currents to produce muscle contractions (Peckham, 1987). The use of FES for gait restoration is limited by the rapid onset of muscle fatigue (Binder-Macleod and Snyder-Mackler, 1993), and powered exoskeletons require batteries and larger actuators to generate the torques necessary to produce the gait motion. However, combining the two technologies may provide the benefits of both powered exoskeletons and FES-based devices and overcome their limitation when used alone. A hybrid device composed of FES and electric motors may have smaller motors because a FES-induced muscle torque would be able to generate a portion of the required torque. The use of a powered exoskeleton in the hybrid device would restrict unwanted degrees of freedom (DOF), reduce stimulation duty cycle of FES, and compensate for FES-induced muscle fatigue. Thus, the hybrid device may be capable of achieving longer walking durations and have additional therapeutic benefits of FES such as muscle growth and increased bone density.

However, multiple motors and FES of lower-limb muscles introduces limb coordination and actuator redundancy (e.g., limb joint torque can be produced by an electric motor of the hybrid exoskeleton and FES of joint flexors and extensors). To date, little research has been done on the design of controllers that consider the actuator redundancy in hybrid neuroprostheses. Quintero et al. (2012) used an adaptive control gain to distribute the control effort between an electric motor and FES for producing knee extensions. A cooperative controller was designed by Ha et al. (2015) for a hybrid walking neuroprosthesis. In this controller, feedback control was used to control the motors to track a desired limb trajectory. Then, an adaptation scheme was used to modify FES profiles to match the joint torque profiles of the electric motors in future gait cycles. However, actuator redundancy was not specifically addressed, this may be because only one or two muscles were stimulated. In Kobetic et al. (2009), a hybrid neuroprosthesis that used implanted electrodes to stimulate 16 muscles was used to achieve walking in a subject with paraplegia. del-Ama et al. (2014b) developed a cooperative control strategy that balanced FES and robotic control of the hybrid neuroprosthesis. The controller used PD control for the electric motors, PID control to maintain support during stance, and an iterative learning controller to develop the stimulation profiles for the swing cycle. The algorithm also detected FES-induced fatigue by measuring decreases in the torque-time integral of the force generated by FES. Currently, these systems only use *ad hoc* finite-state machine controllers and controller stability is not guaranteed.

Given the control challenge, a synergy based closed-loop controller may be ideal to handle the actuators redundancy and high dimensionality in the system. The central nervous system (CNS) is hypothesized to control the largely overactuated musculoskeletal system by activating the individual muscle fibers in groups called synergies, or motor primitives (Sherrington, 1910; Lee, 1984; Grillner, 1985; Tresch et al., 2002; Ting, 2007). Although it is a controversial hypothesis in the field of neural control of movement, these synergies can be thought of as weighted muscle activation patterns for multiple muscles that can be combined to generate coordinated limb movements (e.g., walking or reaching). It is hypothesized that these muscle synergies act as lower dimensional motion primitives that are stored in the spinal cord. Therefore, instead of individually controlling each muscle fiber, the human brain recruits weighted synergies to simplify a task involving multiple muscles or limbs. Currently, synergies are being used for a wide variety of applications, such as musculoskeletal movement analyses or gait therapy (Vinjamuri, 2008; Berniker et al., 2009; Vinjamuri et al., 2010; An et al., 2013; Routson et al., 2013; Steele et al., 2013; Simkins et al., 2014), robot design (Catalano et al., 2012; Wu and Asada, 2014), and control engineering systems (Popovic and Popovic, 2001; Wimbock et al., 2011; Kuppuswamy et al., 2012; Kuppuswamy and Harris, 2014; Wu and Asada, 2014).

From a controls perspective, synergies may be desired for controlling large and complex systems because they can provide simpler lower dimensional controllers that may be more computationally efficient. Some studies have used synergies to achieve lower dimensional control of systems with large DOF. Kuppuswamy et al. (2012) designed a synergy-based feedforward controller to drive robotic systems with redundant actuators to an equilibrium position. In Santello et al. (1998), synergy analyses were used to gain a further understanding of hand postures. This study showed that grasping movements can be explained by the first 2–3 postural synergies. Later, this work was used to design underactuated and simplified humanoid robot hands that mimicked the postural synergies observed in the grasping tasks (Catalano et al., 2012). Synergy inspired controllers were then designed for the humanoid robot hands (Ajoudani et al., 2013). In Berniker et al. (2009), a low-dimensional linear model of non-linear musculoskeletal frog hind-limb is found empirically by using the model order reduction technique balanced truncation. This low-dimensional model is then used to identify a set of muscle synergies using an optimization algorithm which were then used with optimal control techniques to produce a range of movements. The key advantage of synergy-inspired controllers is that the control of complex high DOF systems can be accomplished more efficiently using fewer control signals.

However, synergy-inspired control, to the best of our knowledge, has not been developed for a hybrid walking neuroprosthesis. This work uses the concept of time-invariant synergies and applies them for control design of a hybrid neuroprosthesis. The synergies can be extracted using statistical tools, such as non-negative matrix factorization (NNMF), singular value decomposition (SVD), partial least squares regression (PLSR), or principal component analysis (PCA). Typically, synergy analyses of human motion studies use NNMF. For example, An et al. (2013) used NNMF to analyze muscle synergies of standing up motions with varying seat heights and standing speeds. Steele et al. (2013) studied the impact the number and choice of muscles have on synergy analyses in a musculoskeletal model for an upper extremity task. The benefit of an NNMF algorithm is that it maintains a positive value constraint on the decomposed synergies. This constraint is essential because muscle activations processed from EMG data always have positive values. However, in the hybrid neuroprosthesis system, electric motors are also present, which can generate both positive and negative torque values. Therefore, we employ PCA, instead of NNMF, to avoid the non-negative constraint.

The open hypothesis of this paper is that the hybrid walking system is a better rehabilitative intervention for subjects with spinal cord injury, and a control theoretic result is presented to enable such a system. The key contribution of this paper is the development of an adaptive synergy-based controller for a hybrid neuroprosthesis. Dynamic optimizations were used to produce optimal inputs and gait trajectories, using a subject-specific gait model. A PCA-based decomposition technique was used to extract time-invariant synergies and their activation profiles that were present in the optimal input space. The activation profiles were further adapted online using a gradient-based update law to be used as feedforward control. Then feedback control to the motors was used to improve the performance and robustness of the overall controller. A Lyapunov-based stability analysis was performed to yield semi-global uniformly ultimately bounded tracking. Simulations on a 4-DOF gait model with 10 actuators (FES of three antagonistic muscle pairs, three electric motors, and one walker moment) are presented to depict performance and as a proof-of-concept of the muscle synergy-inspired controller.

## 2 Dynamic Model

**Figure 1:**
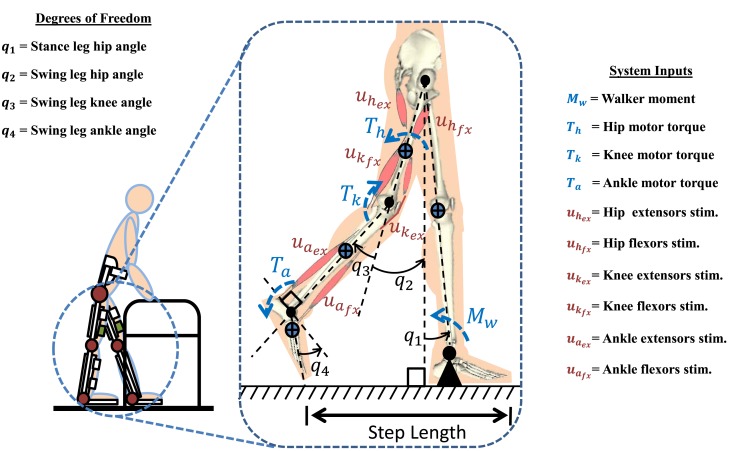
**A four-link gait model based of a subject wearing a hybrid neuroprosthesis while using a walker**. The model has 10 inputs, including FES of six muscles (antagonistic hip, knee, and ankle muscle pairs in the swing leg), three electric motors acting on each joint of swing leg (*T_h_*, *T_k_*, *T_a_*), and a walker moment acting on the stance leg (*M_w_*). The step length is defined as the distance from stance toe to swing toe.

A person taking one step (half of a gait cycle), using a hybrid neuroprosthesis and a walker, is modeled as a four-link musculoskeletal system as seen in Figure 1. The hybrid neuroprosthesis uses a hip knee ankle foot orthosis (HKAFO) that provides kinematic constraints on the user, allowing only motion in the sagittal plane. In addition, the HKAFOs typically use a wrap spring clutch that locks the knee joint of the stance leg to prevent flexion when standing. This reduces the amount of stimulation needed which decreases muscle fatigue and prolongs walking durations (Sharma et al., 2014). The stance leg is modeled as one rigid segment simulating the locking of the knee joint and the ankle is fixed to the ground because only half of the gait cycle is considered in this simulation study. The swing leg has a thigh, shank, and foot segment with three actuators at each joint: motor and FES for flexion and extension of antagonistic muscle pairs. The trunk dynamics were neglected in the model because the use of a walker allows the user to stabilize their truck. The walker is modeled as a moment acting on the stance leg to help propel the body forward and also to keep it upright. The *n*-DOF lower limb model is given as

(1) $$M\left(q\right)\ddot{q}+C\left(q,\dot{q}\right)\dot{q}+G\left(q\right)+f\left(q,\dot{q}\right)+\tau_{d}\left(t\right)+\tau_{\mathrm{ext}}\left(t\right)=\tau ,$$

where *q*, $\dot{q}$, $\ddot{q}\in R^{n}$ are the angular positions, velocities, and accelerations of the leg segments, respectively. In equation (1), *M*(*q*) ∈ ℝ*^n × n^* is the combined inertia of the semi-powered orthosis and human limbs in the swing phase, $C\left(q,\dot{q}\right)\in R^{n\times n}$ is the centripetal/Coriolis matrix, *G*(*q*) ∈ ℝ*^n^* is the gravity vector, $f\left(q,\dot{q}\right)\in R^{n}$ is the viscoelastic vector term that model the passive muscle model, *τ_ext_* ∈ ℝ*^n^* is the torque generated at each joint due to contact with the ground, and *τ_d_* ∈ ℝ*^n^* is any unmodeled effects or disturbances in the system. The torques at the joints are generated by including the musculoskeletal dynamics due to FES (Popović et al., 1999), an electric motor attached at each joint, and the moment generated by the walker force. The torque term is defined as

(2) $$\tau =b\left(q,\dot{q}\right)u,$$

where *b* ∈ ℝ*^n × m^* is the control gain matrix containing the scaling functions for the *m* inputs.

**REMARK 1:** *b*(*q*,$\dot{q}$) and *u*(*t*) are presented for a gait model with DOF, *n* = 4, and control inputs, *m* = 10. However, without loss of generality, the control development and analysis can be extended to *n*-DOF system with *m* inputs.

The model used in this work considers a hybrid neuroprosthesis that uses electric motors and FES via surface electrodes, which non-selectively applies an external voltage potential to a muscle group to generate a contraction. In equation (2), *b*(*q*,$\dot{q}$) and *u*(*t*) ∈ ℝ*^m^* are defined as

(3) $$b={\left[\begin{array}{ccccc} 1 & 0 & 0 & 0\\ 0 & \psi_{h_{\mathrm{fx}}} & 0 & 0\\ 0 & -\psi_{h_{\mathrm{ex}}} & 0 & 0\\ 0 & 0 & \psi_{k_{\mathrm{fx}}} & 0\\ 0 & 0 & -\psi_{k_{\mathrm{ex}}} & 0\\ 0 & 0 & 0 & \psi_{a_{\mathrm{fx}}}\\ 0 & 0 & 0 & -\psi_{a_{\mathrm{ex}}}\\ 0 & \kappa_{h} & 0 & 0\\ 0 & 0 & \kappa_{k} & 0\\ 0 & 0 & 0 & \kappa_{a} & \end{array}\right]}^{T},\;\;u=\left[\begin{array}{cc} M_{w}\\ u_{h_{\mathrm{fx}}}\\ u_{h_{\mathrm{ex}}}\\ u_{k_{\mathrm{fx}}}\\ u_{k_{\mathrm{ex}}}\\ u_{a_{\mathrm{fx}}}\\ u_{a_{\mathrm{ex}}}\\ T_{h}\\ T_{k}\\ T_{a} & \end{array}\right],$$

where subscripts *i* = *h, k, a* stand for hip, knee, and ankle joints of the swing leg. In equation (3), $u_{i_{\mathrm{ex}}},\;u_{i_{\mathrm{fx}}}$ are the stimulation inputs and $\psi_{i_{\mathrm{fx}}},\;\psi_{i_{\mathrm{ex}}}$ are the torque-length and torque-velocity relationships of the flexor and extensor muscles, and *T_i_* is the current input to the motor and the conversion constants (current to torque) of the electric-motor drives is *κ_i_*. The moment due to the walker is denoted as *M_w_*. For this simulation study, hip joint actuation via FES is achieved by stimulating the inner hip muscles (Iliopsoas) for flexion and the Gluteals for extension. Knee joint actuation uses the Quadriceps muscle group for extension and Hamstrings for flexion, and the ankle joint uses the Gastrocnemius for dorsiflexion and Tibialis anterior for plantarflexion.

**ASSUMPTION 1:** The trunk dynamics were neglected in the model because the use of a walker allows the user to stabilize their trunk. However, mass of the head, arm, and torso was incorporated in the model as a point mass.

**ASSUMPTION 2:** The motion is considered only in the sagittal plane because the HKAFO puts kinematic constraints on motion in planes other than sagittal. The HKAFO system uses a wrap spring clutch that locks the knee joint of the stance leg during walking. The stance leg is modeled as one link because the knee is locked and the stance leg ankle acts as an anchor because only half of the gait cycle is considered in this study. These assumptions allow us to model the kinematics of the lower extremities as a four-link chain.

**ASSUMPTION 3:** The walker is used to help produce the required propulsion force or *M_w_*. As the user pushes against the walker to pull themselves forward, the resultant force acts as a moment on the hip of the user or the stance leg, *M_w_*. Therefore, the walker moment, *M_w_*, is treated as an input to the system that can be computed by the developed controller.

**ASSUMPTION 4:** First order muscle activation dynamics are ignored to simplify the control design. This avoids the use of control techniques, such as integrator backstepping (Khalil, 2002), which would add the requirement of additional signals, such as the acceleration, which is typically unavailable or very noisy (Sharma et al., 2009).

**ASSUMPTION 5:** The unmodeled effects or disturbances, *τ_d_*, are bounded as |*τ_d_* | ≤ ϵ_1_ where ϵ_1_ ∈ ℝ^+^ is a constant.

**ASSUMPTION 6:** The control input, *u*, can be decomposed as $u=Wc_{d}+u_{\mathrm{loss}}$, where the synergies in the matrix, *W*, are bounded constants and the time-varying activation coefficients, *c_d_*, are bounded signals. The reconstruction error, *u_loss_*, is bounded by a constant.

## 3 Methods

### 3.1 Dynamic Optimization

Dynamic optimization was used to compute optimal subject-specific gait trajectories and inputs (Kirsch et al., 2013; Sharma et al., 2014). In these optimizations, the model was only restricted to achieve a certain step size and step duration (0.4 m in 75 s). The optimization computes the inputs that minimize a user-defined cost function. One of the benefits of dynamic optimization is that it can account for constraints, such as a limited range of movement and strength of a user. These constraints are accounted for by constraining the optimization to a subject-specific dynamic model. Rather than tracking able-bodied gait data, which may be suboptimal when applied in the case of subjects with paraplegia (Popović et al., 1999, 2003; Dosen and Popovic, 2008, 2009; Pandy and Andriacchi, 2010) and may result in over stimulation of the muscles and quicken the onset of FES-induced muscle fatigue, the dynamic optimizations are used to compute subject-specific optimal trajectories. The following cost function and constraints were used to compute the optimal control inputs and joint angle trajectories:

$$\begin{array}{llll} \underset{\;}{\overset{\min}{u}}\;\;\Pi & =\int_{t_{o}}^{t_{f}}\;u^{T}\mathrm{Qu}\;\mathrm{dt}\; & & \;\\ \text{subject}\;\text{to:} & M\left(q\right)\ddot{q}+C\left(q,\dot{q}\right)\dot{q}+G\left(q\right)+f\left(q,\dot{q}\right)+\tau_{\mathrm{ext}}\left(t\right)=b\left(q,\dot{q}\right)u\; & & \;\\ & q\left(t_{o}\right)=q_{o}\; & & \;\\ & q\left(t_{f}\right)=q_{f}\; & & \;\\ & u\in \left[u_{l},u_{u}\right]\; & & \; \end{array}$$

where *Q* ∈ ℝ*^m × m^* is a symmetric positive definite weight matrix, *q_o_* and *q_f_* are the initial and final joint angle vectors corresponding to the user-defined step length, and the lower and upper bound on the inputs are defined as *u_ℓ_* and *u_u_*. These bounds allow for the computation of an optimal solution while considering the physical constraints of the system, such as the maximum torque a motor can produce or the maximum amount of force a user can produce when using a walker. The inputs to the system are bounded by realistic values. The walker moment was constrained to 100 Nm and the motors torques are constrained to 40 Nm. The optimizations were run with 75 grid points for each control input in *u*. The inputs were interpolated using a linear interpolation. A second-order Heun’s method with a step size of 1 ms was used for numerical integration. This smaller step size was used to prevent numerical divergence that may occur due to the harsh non-linearities in the dynamics, e.g., ground reaction model and passive muscle models, *f* (*q*,$\dot{q}$), which diverge around hyperflexion and hyperextension.

### 3.2 Synergy Extraction

Let *u_d_*(*t*) ∈ ℝ*^m^* be the desired optimal control vector containing desired stimulation and motor voltage levels to achieve the desired optimal trajectory, *q_d_*(*t*) ∈ ℝ*^n^*. The dynamics are written in terms of the optimal control inputs and kinematic trajectories as

(4) $$\begin{array}{llll} & M\left(q_{d}\right){\ddot{q}}_{d}+C\left(q_{d},{\dot{q}}_{d}\right){\dot{q}}_{d}+G\left(q_{d}\right)+f\left(q_{d},{\dot{q}}_{d}\right)+\tau_{\mathrm{ext}}^{*}\left(t\right)\; & & \;\\ & \;\equiv b\left(q_{d},{\dot{q}}_{d}\right)u_{d}\left(t\right),\; & & \; \end{array}$$

where $\tau_{\mathrm{ext}}^{*}$ is the torque created at each joint due to the ground reaction force when using the optimal inputs, and $b_{d}=b\left(q_{d},{\dot{q}}_{d}\right)$ is the desired control gain matrix, which is bounded. By using PCA, the possibly correlated inputs, *u_d_*, can be transformed into linearly correlated inputs, *c_d_*, such as

(5) $$u_{d}=Wc_{d}\left(t\right)+u_{\mathrm{loss}},$$

where *W*  ∈ ℝ*^m × p^* are the precomputed orthogonal synergies, and *c_d_*(*t*) ∈ ℝ*^p^* are the corresponding time-varying activation coefficients of the synergies. The PCA analysis computes *m* synergies that account for all the variability of the data. The synergies are ordered such that the first synergy accounts for most of the variance, the second accounts for the second most, and so on. Typically, the rule of thumb is to use the number of synergies, *p* < *m*, that would account for over 90% of the variability of the data. But since the controller also has feedback control and adapts online, less synergies can be used. Therefore, instead of using three synergies as indicated in Figure 4, only two were used. After dropping the *m* – *p* synergies that account for the least amount of variability in the data, the reconstructed inputs, $Wc_{d}$, do not match the optimal inputs, *u_d_*. Therefore, a reconstruction error, denoted as *u_loss_*, is introduced in equation (5).

### 3.3 Control Development

In this section, we develop a controller that uses the synergies extracted in the previous section to reduce the dimensionality of the feedforward component. Also to improve control performance and robustness, an update law was used to adapt the time-varying activation coefficients online and feedback control for the motors was included. The control objective is to track a continuously differentiable desired trajectory *q_d_* ∈ ℝ*^n^*. The tracking error, *e* ∈ ℝ*^n^*, is defined as

(6) $$e=q_{d}-q.$$

To facilitate the control design and stability analysis, the auxiliary error signal *r* ∈ ℝ*^n^* is defined as

(7) r=ė+αe,

where *α* ∈ ℝ^+^ is a control gain. The closed-loop error is derived by multiplying the time derivative of equation (7) with *M*(*q*) and substituting the dynamics in equation (1) to obtain

(8) $$M\dot{r}=M{\ddot{q}}_{d}+C\dot{q}+G+f+\tau_{d}+\tau_{\mathrm{ext}}-\mathrm{bu}+M\alpha ė.$$

This expression can be written in the form

(9) $$M\dot{r}=-\mathrm{Cr}+Ñ+N_{d}+\tau_{d}+\tau_{\mathrm{ext}}-\mathrm{bu}-e,$$

where $Ñ=N-N_{d}$ and the auxiliary signals *N*(*e,r*) and *N_d_* (*t*) are defined as

$$\begin{array}{llll} N & =M{\ddot{q}}_{d}+C{\dot{q}}_{d}+C\alpha e+G+f+M\alpha ė+e,\; & & \;\\ N_{d} & =M\left(q_{d}\right){\ddot{q}}_{d}+C\left(q_{d},{\dot{q}}_{d}\right){\dot{q}}_{d}+G\left(q_{d}\right)+f\left(q_{d},{\dot{q}}_{d}\right).\; & & \; \end{array}$$

The term *Ñ* in equation (9) can be upper bounded by using the mean value theorem as

(10) $$∥Ñ∥\leq \rho_{1}\left(∥z∥\right)∥z∥,$$

where *ρ*_1_(||*z*||) ∈ ℝ is a positive monotonic bounded function and *z* ∈ ℝ^2^*^n^* is defined as

$$z={\left[\begin{array}{cc} r^{T} & e^{T} \end{array}\right]}^{T}.$$

Note that the auxiliary signal *N_d_* is equal to the left-hand side of the desired muscle synergy dynamics in equation (4), this allows us to substitute $b_{d}u_{d}-\tau_{\mathrm{ext}}^{*}$ in for *N_d_* resulting in

(11) $$M\dot{r}=-\mathrm{Cr}+Ñ+\tau_{d}+{\tilde{\tau }}_{\mathrm{ext}}+b_{d}u_{d}-\mathrm{bu}-e,$$

where ${\tilde{\tau }}_{\mathrm{ext}}=\tau_{\mathrm{ext}}-\tau_{\mathrm{ext}}^{*}$ is the torque due to the ground reaction force mismatch and can be bounded.

**REMARK 2**: Further analysis can be done to show that the bound on ${\tilde{\tau }}_{\mathrm{ext}}$ gets smaller as the position and velocity errors get smaller, i.e., as the tracking errors approach to 0, ${\tilde{\tau }}_{\mathrm{ext}}$ will approach to 0.

By choosing the control law *u* as

(12) $$u=W\hat{c}+\mathrm{kr},$$

where ĉ ∈ ℝ*^p^* is the estimate of *c_d_* and *k* ∈ ℝ*^m × n^* is the feedback gain that is chosen to only influence the electric motors. The estimate of the synergy activation coefficient updates according to the following gradient-based update law with the projection algorithm

(13) $$\dot{\hat{c}}=\mathrm{proj}\left({\dot{c}}_{d}+\Gamma W^{T}b_{d}^{T}r\right),$$

where Γ ∈ ℝ*^p × p^* is a symmetric positive definite learning rate gain matrix. The projection algorithm imposes an upper and lower bound on ĉ, which is used in the stability analysis. More details of this algorithm can be seen in Khalil (2002). The purpose of the adaptation in the activation coefficient is to improve the feedforward component after reconstruction loss and to overcome any system uncertainties. After using equations (5) and (12), equation (11) becomes

(14) $$M\dot{r}=-\mathrm{Cr}+Ñ+\tau_{d}+{\tilde{\tau }}_{\mathrm{ext}}+b_{d}u_{\mathrm{loss}}+b_{d}W\tilde{c}+\tilde{b}W\hat{c}-\mathrm{bkr}-e,$$

where $\tilde{c}\in R^{p}$ and $\tilde{b}\in R^{n\times m}$ are defined as

$$\tilde{c}=c_{d}-\hat{c},\;\;\;\tilde{b}=b_{d}-b.$$

Using the mean value theorem, Assumption 5, and the property of the projection algorithm, the following terms can be bounded as

(15) $$∥\tilde{b}∥\leq \rho_{2}\left(∥z∥\right)∥z∥,\;\;∥W\hat{c}∥\leq \epsilon_{2},\;\;∥{\tilde{\tau }}_{\mathrm{ext}}+b_{d}u_{\mathrm{loss}}∥\leq \epsilon_{3},\;\;∥\tilde{c}||\leq \delta ,$$

where *ρ*_2_(||*z*||) ∈ ℝ is a positive monotonically increasing bounded function and ϵ_2_, ϵ_3_, *δ* ∈ ℝ^+^ are constants.

**THEOREM**: The controller designed in equations (12) and (13) ensures semi-global uniformly ultimately bounded tracking provided that the following gain conditions are met:

$$K_{\min}>\frac{{\left(\rho_{1}\left(∥z∥\right)+\epsilon_{2}\rho_{2}\left(∥z∥\right)\right)}^{2}}{2},\;\;\gamma_{\min}\left\{\mathrm{bk}-\gamma I\right\}>0,$$

where γ*_min_*{⋅} denotes the minimum eigenvalue of a square matrix and *K_min_* ∈ ℝ^+^ is a subsequently defined constant.

***Proof:*** See Supplementary Material.

## 4 Simulation Results

### 4.1 Optimization and Synergy Extraction Results

**Figure 2:**
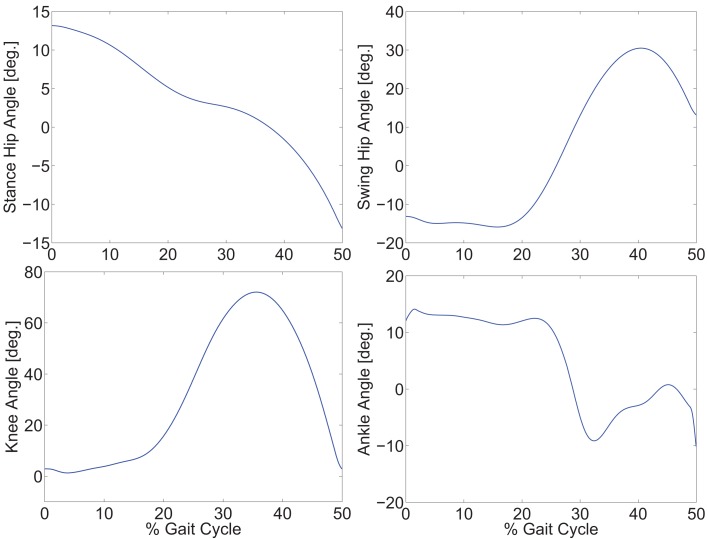
**Optimal gait trajectories for a step size of 0.4 m in 0.75 s**.

**Figure 3:**
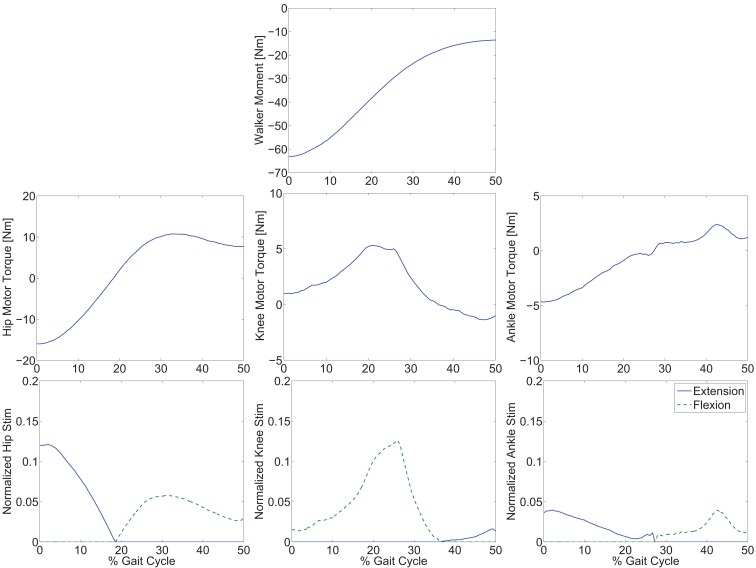
**Optimal inputs to the walker moment, electric motors, and stimulation channels to reproduce the optimal gait trajectories**.

The optimization results are shown in Figures 2 and 3. Figure 2 shows the optimal joint angle trajectories. Figure 3 shows the optimal control inputs. The optimal contributions from the motor and FES can be adjusted by tuning the weights in the cost function.

**Figure 4:**
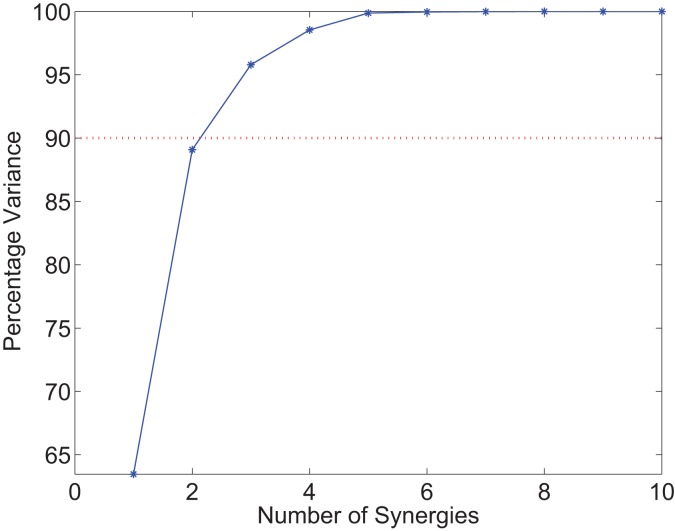
**This plot indicates how much of the data variability would be accounted for based on the number of synergies considered**. Rule of thumb would indicate using three synergies, but since the controller is not solely dependent on the feedforward component less synergies can be used.

**Figure 5:**
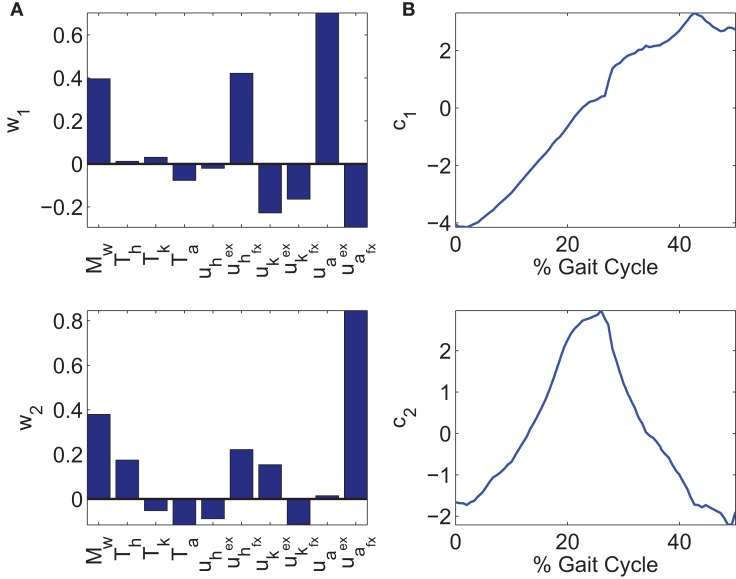
**(A)** Two synergies, *w*_1_ and *w*_2_. **(B)** The corresponding time-varying activation coefficients, *c*_1_ and *c*_2_, of synergies, *w*_1_ and *w*_2_.

The two synergies and their activation profiles extracted through PCA can be seen in Figure 5. Note that the scaling factors in the synergies on the left and the time-varying activation coefficients on the right can have negative values. This makes it harder to interpret what influence each synergy has on the system, but it is unavoidable when PCA is used. Also in the optimizations, the inputs to the stimulation channels are constrained to be positive, but after extracting the synergies, this property was lost. This results in negative stimulation values that are not applicable with FES because muscles are unidirectional actuators. Therefore, when implementing the controller, any negative stimulation inputs were set to 0.

### 4.2 Tracking Results

The newly developed controller in equation (12) was simulated on a four-link rigid body gait model, developed in SimMechanics [MathWorks, CA, USA]. The head, arms, and torso were modeled as a point mass at the hips. The stance leg was modeled as a single link with a fixed knee joint and a pinned ankle joint. The swing leg was modeled with four-links: thigh, shank, and foot. Each link in the swing leg had redundant actuation, i.e., an electric motor and FES for the muscle flexors and extensors. The influence of the walker was modeled as a moment acting on the stance leg. This moment was used to help propel the body forward and help keep the body stable and upright. The unmodeled effects or disturbances, *τ_d_*, was incorporated by injecting uniformly distributed noise into the four joints. The masses and lengths for each limb were taken from anthropometric data (Winter, 2009), and the muscle parameters of a subject with SCI were taken from Popović et al. (1999) and Dosen and Popovic (2009). The ground reaction force was realized on two contact points: the toe and heel. The model uses a spring-damper system in the vertical direction and a static or kinetic friction model in the horizontal direction when the foot is in contact with the ground. More information on the specifics of this ground reaction model can be found in Geyer and Herr (2010).

**Figure 6:**
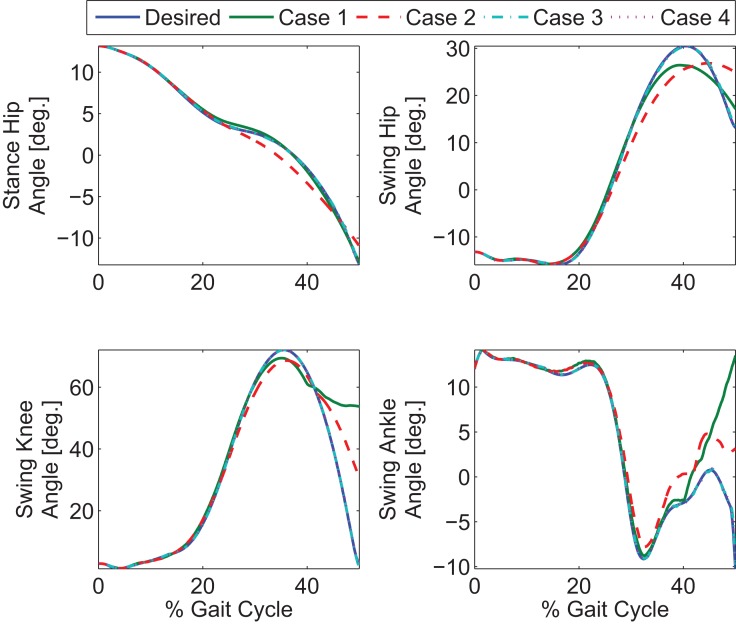
**Four cases for gait control using a hybrid neuroprosthesis**. Case 1 only used the feedforward synergies, Case 2 used the adapted feedforward synergies, Case 3 considered both the adapted feedforward synergies and feedback control, and Case 4 used the full optimal inputs and feedback control. Note that the profile from the third and fourth cases almost perfectly overlaps the desired profiles.

**Figure 7:**
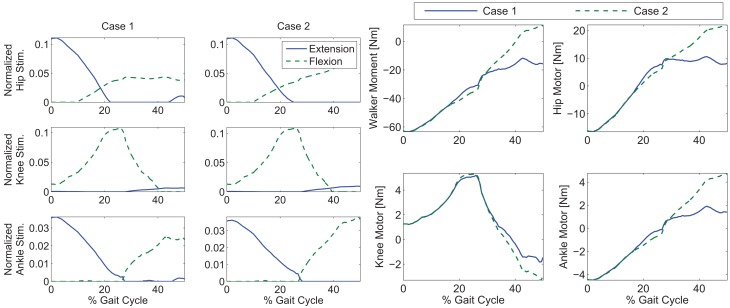
**Control inputs for Cases 1 and 2 of the simulations**. Note that the control input profile shapes, after PCA decomposition, in Case 1 may not be similar to the optimal inputs in Figure 3.

**Figure 8:**
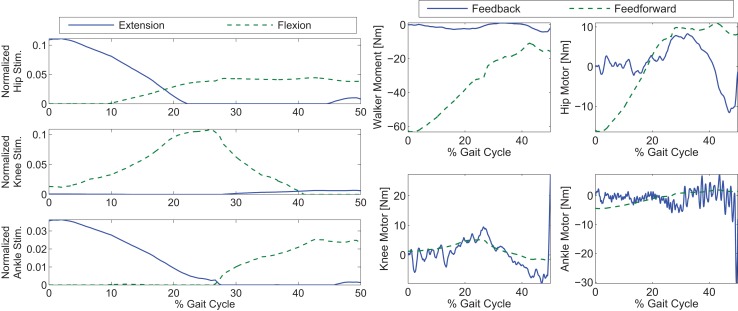
**Control inputs for Case 3 of the simulations**. The feedback’s contribution was used only in the walker moment and motor torques.

**Figure 9:**
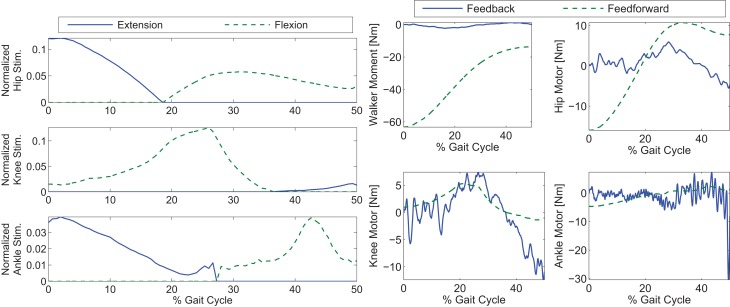
**Control inputs for Case 4 of the simulations**. The feedback’s contribution was used only in the walker moment and motor torques.

**Figure 10:**
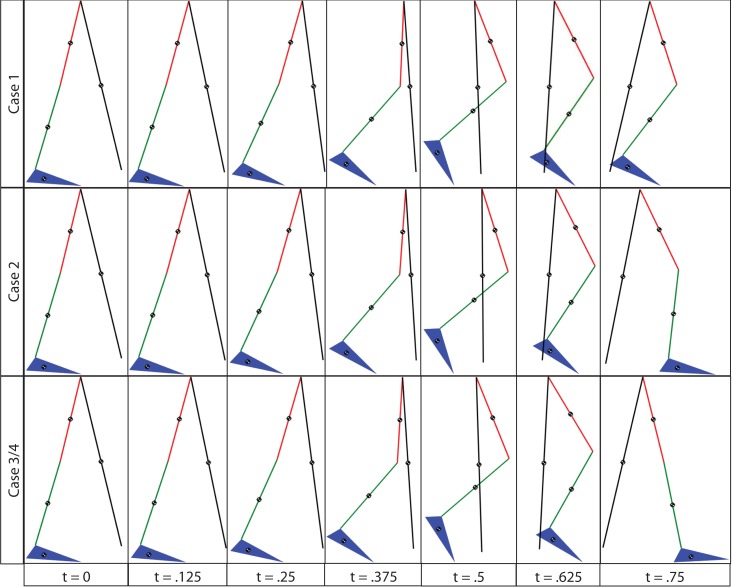
**The gait sequence for the four cases, a step length of 0.4 m with a step duration of 0.75 s was used**. Since the errors for the third and fourth cases are so close their gait sequences look identical.

To explore the efficacy of the controller, the simulations were done with four cases. Case 1 considered the synergies as the feedforward component but with no adaptation, i.e., $Wc_{d}$ in equation (5). Case 2 considered the synergies with adaptation, i.e., *Wĉ* with the adaptive law in equation (13). Case 3 considered both the synergies with adaptation and feedback control, i.e., equations (12) and (13). Case 4 considered the full optimal inputs computed in the optimizations with feedback control. Only the motors and walker moment were used as effectors to provide feedback. The control gains used in the cases that included feedback control were *k* = 10 and *α* = 100. In the two cases where adaptation was present, the learning rate used for the two synergies were 0.0175 and 0.001. The results are shown in Figures 6–10. The root mean squared error (RMSE) for the four joints for each case can be seen in Table 1. Of all the cases, the third and fourth cases were found to provide the best performance. In the first case, the feedforward component provides just enough control input to produce the movements but fails to clear the ground to complete the step. This is because the toe makes contact with the ground model early and begins to drag. In the second case, the swing leg joint angles match the desired profiles better and almost complete the walking step but the swing foot does not reach the floor in the allotted time of 0.75 s. In the third and fourth cases, the trajectories match the desired profiles almost perfectly.

**Table 1:** **The root mean squared (RMS) error for the four simulated cases**.

Case	RMS error (°)			
	Stance hip	Swing hip	Swing knee	Swing ankle
1	0.30	1.78	11.53	3.86
2	0.90	3.43	7.22	2.39
3	0.09	0.22	0.15	0.14
4	0.07	0.09	0.14	0.08

*Case 3 that had two synergies and feedback and Case 4 that had full optimal feedforward and feedback had the smallest RMS errors, followed by Case 2 that had just adaptive feedforward, and then Case 3 with the non-adaptive feedforward*.

## 5 Discussion

A muscle synergy approach can be useful for engineered systems with redundancy in effectors. For example, the research by de Rugy et al. (2013) mentions the usefulness of muscle synergies in FES-based systems. The muscle synergy principle has also been suggested as a hierarchical control framework for redundant manipulators (Todorov et al., 2005; Artemiadis and Kyriakopoulos, 2010), brain–machine interface-based control (Vinjamuri et al., 2011), and for the design and control of a humanoid robotic hand (Cho et al., 2007; Rosmarin and Asada, 2008; Catalano et al., 2012; Grioli et al., 2012). In our proposed adaptive control scheme, we showed that the synergy-based approach can be modified to provide a lower dimensional feedforward controller and combined with a feedback controller to control a hybrid walking neuroprosthesis.

As shown in the simulations, the new controller (Case 3) performs as expected only when both the adaptive feedforward and feedback components were active. However, in Case 1, when two synergies were used alone, the key characteristics of the optimized gait was reconstructed, but the inputs from the two synergies were not enough to clear the ground and complete a full step as can be seen in Figure 10. This was likely caused by the reconstruction error, *u_loss_* in equation (5), due to the PCA decomposition. Evidence of this can be seen by comparing the optimal inputs in Figure 3 and the feedforward inputs in Case 1, as shown in Figure 7. To overcome the reconstruction error due to the synergy decomposition, we proposed adding an adaptive component and a feedback component to the synergy controller. In Case 2, the adaptive synergies provided sufficient control inputs to complete the walking step as well as enable the foot to clear the ground during the swing phase but the swing knee joint angle does not end at 0° as seen in Figure 10. This is evident in Figure 6, near the 40% gait cycle region, where the swing hip, knee, and ankle profiles showed improved tracking of the desired profile. In Case 3, the feedback control to the motors further improved the performance and the actual gait trajectories tracked the desired profile almost perfectly. In this case, the adaptive feedforward control may have given an approximate desired control input, and the feedback control fine tuned the input to further minimize the error.

In Figure 8, it can be seen that the amount of feedback motor torque and feedforward motor torque are comparable in magnitude. This indicates that the feedback is not doing all the work in this case. The need for the feedback torque is necessary because after dimensionality reduction, the feedforward component may not be enough to reproduce the movement due to reconstruction loss. However, in Case 4’s results (Figure 9), where optimal inputs instead of reconstructed inputs were used, it can be seen that feedback control still played the same role as it did in Case 3. This is because the optimizations that computed the feedforward components did not consider system disturbances.

It can then be concluded that even if one were to use more synergies (greater than two), the feedforward component would still not be enough. But the benefit of decomposing the optimal inputs and truncating the amount of synergies used is the reduced amount of data needed in the real-time implementation of the controller. That is to say, instead of having the 10 signals with 750 data points each (0.75 s at 1 kHz) from the optimal inputs, the feedforward controller uses two signals and a matrix *W*  ∈ ℝ^2×10^ in this case. Therefore, the feedforward component was reduced from using 7500 data points to using only 1520 data points.

The limitation of PCA is that the decomposed synergies may not be easily interpreted. For example, in each synergy there is a scaling factor for each of the *m* control inputs and some synergies may have negative scaling factors. A negative scaling factor may not have any physical meaning (e.g., the stimulation inputs are always positive). Also, adaptation in one activation coefficient changes the scaling factors of all the control inputs in the corresponding synergy, which may result in a non-gait like motion. Interpretation of the synergies becomes even more inscrutable when PCA results in activation coefficients that can be negative.

The new control development is based on time-invariant synergies, which means that all the inputs within a synergy set were activated synchronously and temporal delays were not considered. Perhaps, the use of time-varying synergies, which have a spatial and temporal component, would result in less synergies and a more effective feedforward component. Also, synergies specific to the optimized gait data were extracted which means that they may not span the full input space of the system. However, the developed controller is general enough to be implemented on larger systems with more degrees of freedom and may be used with any set of synergies. A general set of synergies that are applicable to multiple tasks/movements, such as different step lengths and gait speeds, sitting/standing, or ascending/descending stairs, would provide a comprehensive data set to accomplish a control design for the hybrid neuroprosthesis. An optimization algorithm, such as the one used in Berniker et al. (2009), may be used to extract a more general set of synergies from a reduced model (lower dimensional) and used with this controller for a general task. While the focus of this paper was on designing automatic control methods that can handle actuator redundancy, gait optimizations in our result can be improved by using high fidelity gait models or optimization methods, such as in Ackermann and van den Bogert (2010). Our future work will explore extracting muscle synergies based on optimization of these high DOF models and implementing these controllers on human subjects.

One method to find a generalizable set of synergies would be to have the optimizations compute a common set of synergies and the time-varying activation profiles to achieve multiple walking speeds and other tasks directly as opposed to computing the optimal inputs then extracting the synergies. The resulting set of synergies may be more generalizable than the current set extracted through PCA. Another benefit of using optimizations to extract the synergies is the possibility of more restrictions, e.g., non-negative scaling factors in synergies (a limitation of PCA) or non-negative stimulation after synergy transformation.

In order to test these types of controllers in experimental trials, the controller must be scaled-up to achieve motions other than gait, such as sitting and standing. This can be achieved by designing a library of synergies that encompass walking, sitting, and standing. Also, because these optimizations are model based, extensive system identification experiments are required to find the subject-specific parameters that are used in the models. Undoubtedly, validating the synergy-inspired controller is not completely feasible with surface FES because the hip flexors and extensors are harder to access. However, during experimental implementation of this controller, only a motor could be used at the hip joint while muscle synergy-inspired controller can still be verified for redundant actuation at the knee and ankle joints. Alternatively, an invasive FES system, such as in Triolo et al. (1996), may provide access to over 40 different lower-limb muscles. Therefore, a synergy-inspired controller may be a very good candidate for the hybrid neuroprosthesis system proposed in Kobetic et al. (2009).

## 6 Conclusion

In this paper, an adaptive synergy-based controller was presented for a hybrid walking neuroprosthesis. The controller used optimal inputs and trajectories, computed from dynamic optimizations, that were performed on a subject-specific gait model. A PCA algorithm was used to extract synergies from the optimal inputs to be used as a feedforward component to the controller. An update law was derived, using Lyapunov stability analysis, to adapt the time-varying activation coefficient of the synergies online. In addition, a feedback PD controller was used to make the controller more robust to disturbances. The efficacy of the controller was demonstrated in simulations on a four-link gait model with 10 actuators, including a walker moment, electric motors, and FES of the muscle flexors and extensors. Future work will focus on using time-varying synergies and different adaptation schemes, such as the adaptation of the scaling factors in the synergies.

## Conflict of Interest Statement

The authors declare that the research was conducted in the absence of any commercial or financial relationships that could be construed as a potential conflict of interest.
