# Supplementary material for: A Muscle Synergy-Inspired Adaptive Control Scheme for a Hybrid Walking Neuroprosthesis
Source: Front Bioeng Biotechnol. 2015 Dec 21;3:203. doi: 10.3389/fbioe.2015.00203 (PMC4685894; doi:10.3389/fbioe.2015.00203)
Supplement: Supplementary file 1 [file datasheet_1.pdf]

Supplementary Material: Stability Analysis

N. Alibeji[1], N. Kirsch[1], and N. Sharma[1]

[1]*Department of Mechanical Engineering and Materials Science, University of Pittsburgh, Pittsburgh, PA; Email: {nak65, naa33, nis62}@pitt.edu*

Appendix

Proof: A continuously differentiable Lyapunov candidate $U(z,t) \in \mathbb{R}$, is defined as

$$U = \frac{1}{2}e^T e + \frac{1}{2}r^T M r + \frac{1}{2}\tilde{c}^T \Gamma^{-1}\tilde{c}. \tag{A.1}$$

The Lyapunov candidate U can be upper and lower bounded as

$$\lambda_1 \|z\|^2 \leq U \leq \lambda_2 \|z\|^2 + \Upsilon, \tag{A.2}$$

where $\Upsilon,\ \lambda_1,\ \lambda_2 \in \mathbb{R}^+$ are constants.

Taking the time derivative of $U(z,t)$ and using (7) and (14) results in

$$\begin{aligned}\dot{U} = {} & e^T(r - \alpha e) + \frac{1}{2}r^T \dot{M} r + \tilde{c}^T \Gamma^{-1}\left(\dot{c}_d - \dot{\hat{c}}\right) \\ & + r^T\left(-Cr + \tilde{N} + \tau_d + \tilde{\tau}_{ext} + b_d u_{loss} + b_d W \tilde{c} + \tilde{b} W \hat{c} - bkr - e\right).\end{aligned}$$

After using the skew-symmetry property [1] and canceling out the like terms, the previous equation becomes

$$\begin{aligned}\dot{U} = & -\alpha e^T e - r^T bkr + \tilde{c}^T \Gamma^{-1}\left(\dot{c}_d - \dot{\hat{c}}\right) \\ & + r^T\left(\tilde{N} + \tau_d + \tilde{\tau}_{ext} + b_d u_{loss} + b_d W \tilde{c} + \tilde{b} W \hat{c}\right).\end{aligned}$$

Using the update law in (13) yields

$$\dot{U} = -\alpha e^T e - r^T bkr + r^T\left(\tilde{N} + \tau_d + \tilde{\tau}_{ext} + b_d u_{loss} + \tilde{b} W \hat{c}\right).$$

The previous equation can be bounded using (15) and Assumption 4 to get

$$\begin{aligned}\dot{U} \leq & -\alpha e^T e - r^T bkr \\ & + \|r\| \left[\left(\rho_1(\|z\|) + \epsilon_2 \rho_2(\|z\|)\right)\|z\| + \epsilon_1 + \epsilon_3 \right].\end{aligned}$$

Using nonlinear damping to separate the terms and further bounding results in

$$\dot{U} \leq -\alpha e^T e - r^T (bk - \gamma I) r + \frac{(\rho_1(\|z\|) + \epsilon_2\rho_2(\|z\|))^2 \|z\|^2}{2\gamma} + \frac{(\epsilon_1 + \epsilon_3)^2}{2\gamma},$$

where $\gamma \in \mathbb{R}^+$ is a constant. This expression can be bounded as

$$\dot{U} \leq -\left(K_{min} - \frac{(\rho_1(\|z\|) + \epsilon_2\rho_2(\|z\|))^2}{2\gamma}\right)\|z\|^2 + \frac{(\epsilon_1 + \epsilon_3)^2}{2\gamma}, \tag{A.3}$$

where K_{min} is defined as $K_{min} = min\{\alpha, \gamma_{min}\{bk - \gamma I\}\}$. Consider a set $\mathbb{S}$ defined as

$$\mathbb{S} \triangleq \left\{ z(t) \in \mathbb{R}^{2n} \mid \|z(0)\| < \sqrt{\frac{\lambda_1}{\lambda_2}\left(\overline{\rho}^{-2}\left(\sqrt{2\gamma K_{min}}\right) - \frac{\Upsilon}{\lambda_1}\right) - \frac{B\lambda_2}{\delta}} \right\},$$

where $\overline{\rho}$ is a positive monotonically increasing bounded function defined as $\overline{\rho} = \rho_1(\|z\|) + \epsilon_2\rho_2(\|z\|)$, and $B \in \mathbb{R}^+$ is a subsequently defined constant. In $\mathbb{S}$, $A(\|z\|)$, which is defined as $A(\|z\|) = K_{min} - \frac{(\rho_1(\|z\|)+\epsilon_2\rho_2(\|z\|))^2}{2\gamma}$, is bounded by a constant $\delta \in \mathbb{R}^+$as

$$A(\|z\|) \geq \delta.$$

Adding and subtracting $\frac{\delta}{\lambda_2}\Upsilon$ to (A.3) and using (A.2), (A.3) becomes

$$\dot{U} \leq -\frac{\delta}{\lambda_2} U + B, \tag{A.4}$$

where $B = \frac{\delta}{\lambda_2}\Upsilon + \frac{(\epsilon_1+\epsilon_3)^2}{2\gamma}$. (A.4) can be integrated with respect to time to obtain

$$U(z,t) \leq U(0) e^{-\frac{\delta}{\lambda_2}t} + \frac{B\lambda_2}{\delta}\left(1 - e^{-\frac{\delta}{\lambda_2}t}\right). \tag{A.5}$$

From (A.5) it is evident that $U(z,t)$ decays exponentially to a bound $\frac{B\lambda_2}{\delta}$ which can be minimized using the control gains. Therefore, it can be concluded that $U \in \mathcal{L}_\infty$ and the states $e,\ r \in \mathcal{L}_\infty$. Further analysis can be done to show that the $\|z\|$ decays to the ball of radius $\sqrt{\frac{B\lambda_2}{\delta\lambda_1}}$. By Theorem 4.18 in [1], we can conclude that the origin of z is semi-global uniformly ultimately bounded (SGUUB). ■

References

[1] H. K. Khalil, *Nonlinear Systems*, 3rd ed. Prentice Hall, 2002.
